# Electrical Detection of Magnetic Spin Textures Using Pure Spin Currents in Graphene

**DOI:** 10.1021/acs.nanolett.5c04846

**Published:** 2025-12-21

**Authors:** Lars Sjöström, Bing Zhao, Maha Khademi, Roselle Ngaloy, Alexei Kalaboukhov, Johan Åkerman, Saroj P. Dash

**Affiliations:** † Department of Microtechnology and Nanoscience, Chalmers University of Technology, SE-41296 Gothenburg, Sweden; ‡ NanOsc AB, SE-16440 Kista, Sweden; ¶ Department of Physics, 3570 University of Gothenburg, SE-41296 Gothenburg, Sweden; § Center for Science and Innovation in Spintronics, Tohoku University, 2-1-1 Katahira, Sendai 980-8577, Japan; ∥ Research Institute of Electrical Communication, Tohoku University, 2-1-1 Katahira, Sendai 980-8577, Japan; ⊥ Wallenberg Initiative Materials Science for Sustainability, Chalmers University of Technology, SE-41296 Gothenburg, Sweden; # Graphene Center, Chalmers University of Technology, SE-41296 Gothenburg, Sweden

**Keywords:** magnetic domains, spin texture, all-electrical detection, sensing, spin-valve, graphene

## Abstract

Electrical creation, control, and detection of magnetic spin textures are pivotal in the advancement of magnetic data storage and logic technologies. All-electrical methods are especially important for detecting such magnetic textures due to their easier implementation in electronic circuits. Here, we demonstrate the electrical detection of different magnetic multidomain and vortex patterns through pure spin-polarized electronic transport in graphene spin-valve devices. We utilized ferromagnetic electrodes with engineered magnetic domain patterns to inject a spin current into the graphene spin transport channel, which was subsequently detected by a reference ferromagnetic electrode with a quasi-single domain. Distinct multilevel spin-valve switching patterns facilitate the discernment of the spin polarization from the diverse domain patterns. The magnetization of the ferromagnetic electrodes is correlated with magnetic force microscopy and micromagnetic simulations. These developments open avenues for probing magnetic domain and spin dynamics through fully electrical means with pure spin currents for spintronic memory and logic.

## Graphical abstract

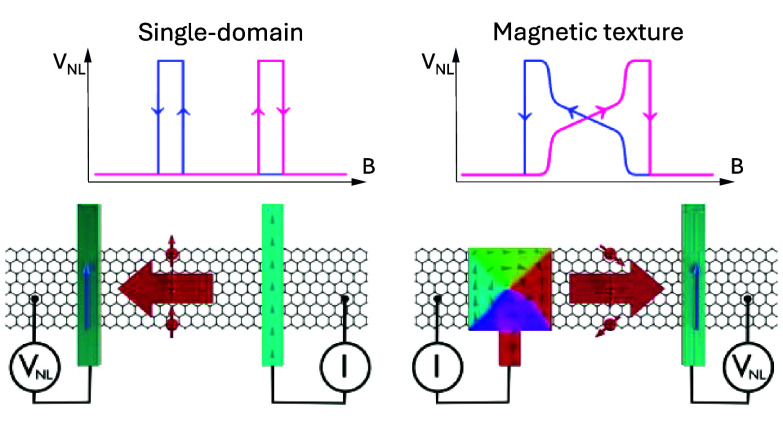

Magnetic domains and spin textures have attracted a significant interest due to their great promise for novel technological applications such as magnetic racetrack,^1,2^ magnetic tunnel junction^3^ and magnetic domain-wall^4,5^ memories, probabilistic^6^ and neuromorphic^7,8^ computing, as well as magnetologic devices.^9,10^ Among the main advantages of using magnetic textures for the storage and processing of data are their nonvolatility and fast dynamics, opening avenues for a new generation of fast and energy-efficient computing. For many of these applications, accurate creation, manipulation and detection of different magnetic textures are of paramount importance.

For detection of different magnetic textures, a variety of different microscopy techniques, such as magnetooptical Kerr effect microscopy,^2,5,10,11^ magnetic force microscopy,^1,10,12,13^ spin-polarized scanning tunneling microscopy,^14^ Lorentz transmission electron microscopy^15^ and nitrogen-vacancy magnetometry,^16^ have been utilized in different magnetic materials. However, for practical applications, all-electrical detection of magnetic textures is preferable due to its compatibility with conventional electronic devices and its potential for more straightforward down-scaling. Such all-electrical detection of magnetic textures has been demonstrated via magnetoresistance,^17,18^ spin Hall effect,^19^ anomalous Hall effect,^18,20^ magnetic tunnel junction^3,21–23^ and metallic spin-valve^24–27^ measurements. Importantly, spin-valve measurements have the major advantage that they can be performed in nonlocal geometries utilizing pure spin currents for detection of magnetic textures, thereby avoiding effects from charge currents on the measured spin signal.

However, such detection of magnetic textures by nonlocal spin-valve measurements have so far predominantly been performed using devices with metals such as copper as the spin transport channel material.^24–27^ Due to the short spin diffusion lengths in metals at room temperatures, these measurements have been limited to cryogenic temperatures up to 77 K.^24–27^ In contrast, graphene has been demonstrated as an excellent spin transport material with long spin diffusion lengths and robust spin transport up to room temperature,^28–35^ and has successfully been utilized for spin-valve devices with a broad range of different materials^36–39^ and spin logic circuits.^40,41^ Such graphene spin circuits have furthermore been proposed to be useful for programmable spin-based computing, where electrical detection and processing of signals from magnetic states are required.^8,42,43^

Here, we demonstrate the electrical detection of magnetic spin textures of a ferromagnet through pure spin-polarized electronic transport in graphene spin-valve devices at room temperature. Using quasi-single-domain, multidomain and vortex magnetic textures, we demonstrate the discernment of the spin polarization contributed by the distinct magnetic domain patterns. The measured spin-valve signals are correlated with magnetic force microscopy and micromagnetic simulations of the spin textures in the ferromagnetic (FM) electrodes. These advancements provide valuable understanding in detecting magnetic domain configurations via electrical methods using graphene spin-valve devices, paving the way for integrating magnetic spin textures in graphene spin circuits.

Graphene spin valve devices with a series of specially shaped FM cobalt electrodes were fabricated and measured, as illustrated schematically in Figure 1a and shown with a representative device in Figure 1b. The device fabrication was performed utilizing chemical vapor deposition (CVD) graphene and scalable fabrication processes. The geometric shapes of the ferromagnets (FMs) impact their magnetization dynamics in the presence of an external magnetic field, which can be traced by spin transport measurements. A charge current *I* was passed between the injector FM electrode and a nonmagnetic (NM) reference, generating spin injection from the FM into the graphene channel. The spins then diffused along the graphene channel and could be detected as a nonlocal voltage *V*_*NL*_ between the detector FM contact and a NM reference outside of the charge-current loop, thereby avoiding charge-current effects in the measured signal. As an external magnetic field *B*_*y*_ was swept along the easy axis of the FM contacts, the measured spin signals traced the changing magnetization of the FM contacts, where a low (high) nonlocal voltage corresponds to a parallel (antiparallel) magnetization configuration between the injector and the detector contacts. Whereas the elongated rectangular electrodes contain quasi-single-domain magnetic textures that switch in single steps and give rise to typical rectangular spin valve signals, the specially shaped contacts exhibit more complex magnetic-texture behaviors and generate multilevel spin-valve switches, as illustrated in Figure 1c. This relationship between spin texture and detected spin signal can be visualized as in Figure 1d, where a nonuniformly changing net magnetization in the contact gives rise to a correspondingly uneven nonlocal voltage.

**Figure 1:**
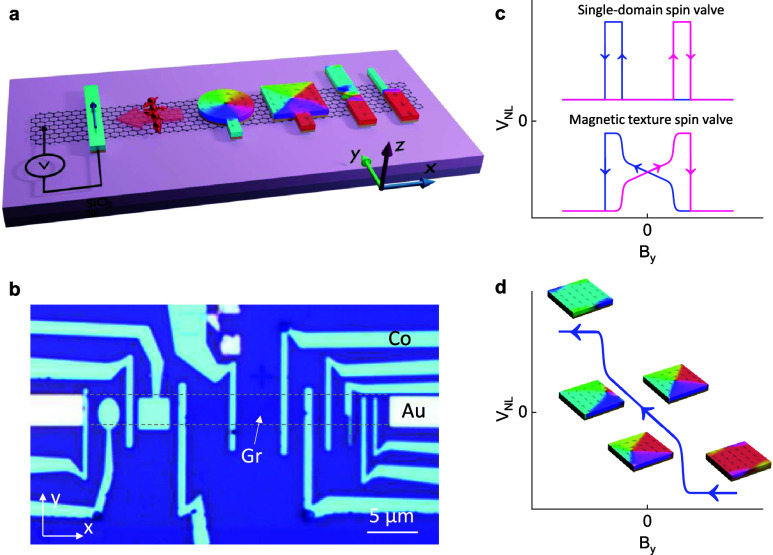
Graphene spin-valve device for electrical detection of magnetic spin textures. (a, b) Schematic (a) and optical microscope image (b) of a representative device used for electrical measurements of different magnetic spin textures in cobalt using a graphene spin valve. A nonlocal measurement geometry was used, where a pure spin current is passed between an electrode with a special magnetic texture and an electrode with a quasi-single magnetic domain. The dashed lines in (b) indicate the borders of the graphene spin transport channel. (c) Schematics of expected nonlocal spin signals for spin-valve devices with single-domain (top) and magnetic-texture (bottom) FM electrodes. (d) Schematic of the expected spin signal contribution from a vortex spin texture, as well as the corresponding magnetic textures of a square electrode at different stages of the magnetic field sweep.

A standard spin-valve signal for quasi-single-domain contacts (Figure 2a) is shown for reference in Figure 2b, where an expected switching signal is observed for parallel and antiparallel alignments of the respective magnetizations of the FM contacts.^32–34^ However, in the devices with FM **“single-notch”** and **“double-notch”** contacts (Figure 2c,e), magnetic domain walls are pinned by the notches.^1,13,17,44^ In this way, the contacts are decoupled into separate magnetic domains that switch in sequence when the external magnetic field is increased. Analogously, in the device with a **“stepped”** contact (Figure 2g), the wider and the narrower sections of the contact are separated by a pinned domain wall at the sharp step geometry.^5,45^ The multidomain magnetization dynamics are seen as subsequent steps of increasing *V*_*NL*_ in the spin-valve signals in Figure 2d,f,h, where the relative amplitude of the signal indicates the proportion of the FM contact that has been switched. These multidomain contact designs can be extended to realize any number of magnetic domains and intermediate magnetic states, which would be distinguishable in such a graphene spin-valve device. Given the noise level of the present proof-of-concept devices, it is estimated that net magnetization changes of down to ∼ 7% can reliably be detected, but this resolution can be dramatically enhanced through fabrication optimization and integration of hBN tunnel barriers to improve the spin injection efficiency between the FM contacts and the graphene channel.^46–48^ The experimental results show good qualitative agreement with MuMax3 micromagnetic simulations,^49–51^ as shown with distinct magnetic textures for different points along the *B*_*y*_ sweeps in the insets of Figure 2b,d,f,h as well as with magnetic hysteresis loops in Supplementary Figure 1–2, which is discussed further in Supplementary Note 1.

**Figure 2:**
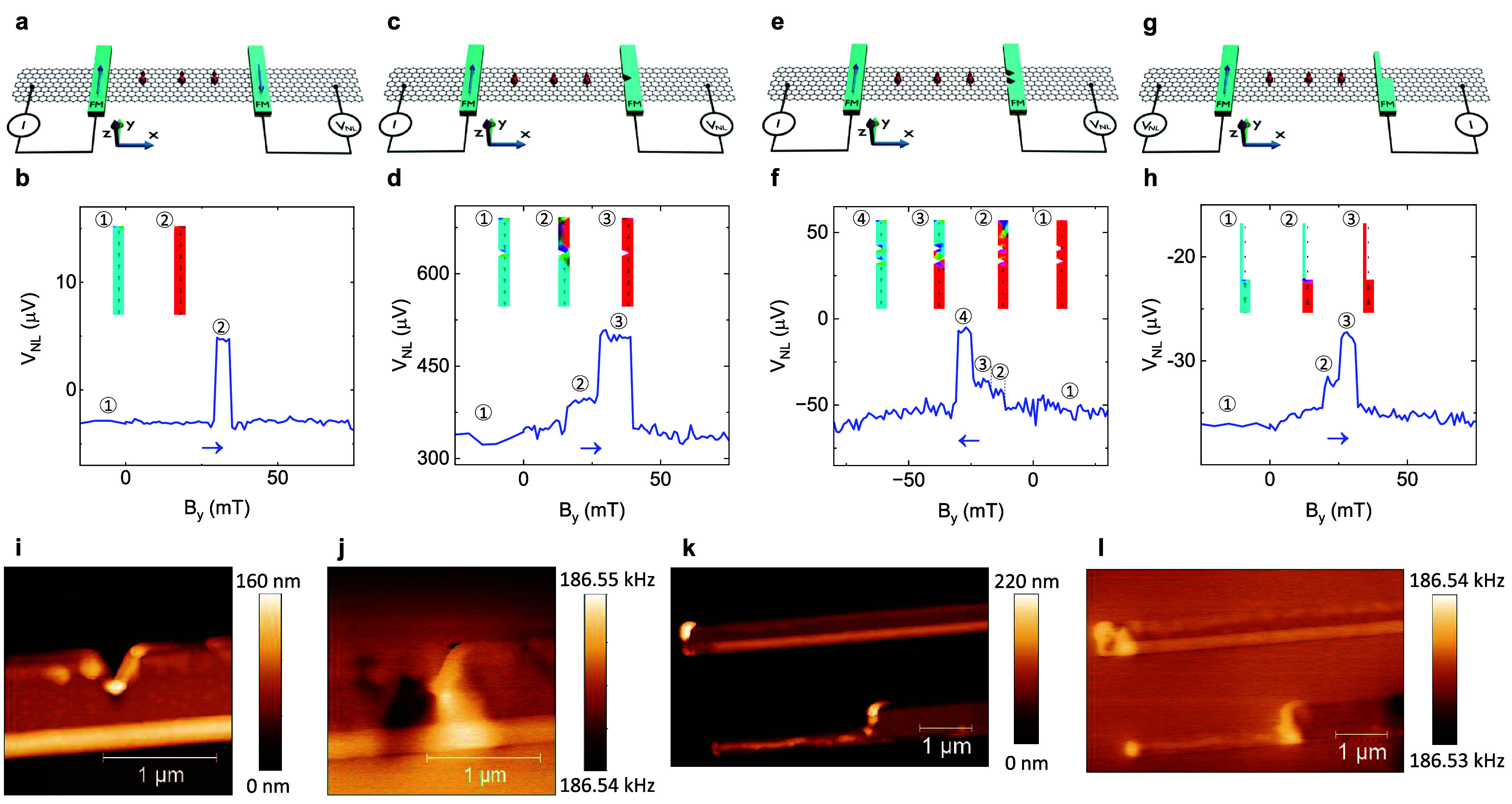
Detection of multidomain magnetic textures. (a, c, e, g) Schematics of spin-valve devices with two rectangular FM contacts (a) and with a single-notch (c), a double-notch (e), and a stepped (g) FM contact. (b, d, f, h) Spin-valve signals of the devices in (a, c, e, g), respectively. The arrows indicate the *B*_*y*_ sweep directions. Forward and backward sweeps for (b, d) are shown in Supplementary Figure 3. Insets: Simulated magnetic textures illustrating the magnetization switching of the respective FM contacts. The circled numbers indicate the correlation between the magnetic textures and the spin-valve signals. (i, j) AFM topography (i) and MFM frequency contrast (j) images of a notch in a FM contact. (k, l) AFM topography (k) and MFM frequency contrast (l) images of a rectangular (top) and a stepped (bottom) FM contact.

The multidomain magnetization of the notched and stepped contacts was verified using magnetic force microscopy (MFM). Figure 2i-l show atomic force microscopy (AFM) and MFM images of a notched, a rectangular and a stepped contact, respectively, where the MFM frequency contrast comes from the second gradient of the magnetic force, which can be related to the magnetization direction. The MFM signal is mostly homogeneous along the length of the electrodes, but pinned magnetic domain walls are clearly visible as adjacent dark and bright areas at the notch in Figure 2j and as a bright area at the sharp step geometry in Figure 2l. Similar MFM images with pinned magnetic domain walls could be recreated from the simulated magnetic textures, as presented in Supplementary Figure 1–2. Both the micromagnetic simulations and the MFM measurements hence agree very well with the observed multistep magnetic dynamics in the spin-valve measurements.

In order to probe magnetic vortex textures, a spin-valve device with a **circular** FM contact (illustrated in Figure 3a) was investigated. Due to its symmetric shape, the circular contact has an ill-defined magnetic easy axis, and its magnetization is not expected to be contained to purely along the *y* axis. As a testament to the more complex magnetic texture, spin-valve measurements of the device with a circular contact indicate a different type of dynamics compared to those of the more elongated devices above. As shown in Figure 3b, the spin-valve signal does not only contain sharp steps in *V*_*NL*_, but also a gradual slope. The magnetization dynamics can be understood as follows. For a large positive field *B*_*y*_, the magnetization of the circular contact is saturated in the +*y* direction. When the magnitude of the field is reduced below a certain level, a magnetic vortex is created close to the left side of the circular contact, which reduces the net magnetization of the contact and is seen as a sharp step in the spin-valve signal. Interestingly, this happens before the applied field reaches zero, indicating that the vortex texture is the ground state of the circular contact’s magnetization.^15,52,53^ As the external magnetic field is subsequently swept toward the −*y* direction, the vortex moves continuously toward the right side of the FM contact, which gradually increases the −*y* net magnetization of the contact. Since the spin-valve measurements are only sensitive to spins along the *y* direction (i.e., the easy axis of the FM detector contact), this leads to a gradual increase in *V*_*NL*_. The small spikes during this gradual slope may come from pinning of the magnetic vortex as well as electrical noise. To be noted, while the presence and continuous movement of the magnetic vortex can be tracked by the spin-valve measurement, the vortex’ chirality cannot be discerned. Finally, at a sufficiently large negative *B*_*y*_, the vortex is annihilated and the contact magnetization is saturated in the −*y* direction, which is seen as the second sharp step in the spin signal. These magnetization dynamics were replicated by simulations, and are illustrated by five representative simulated spin textures in the inset of Figure 3c and as an animation in Extended Data 1. Further, the conformity between the experimental spin signal and the simulated net *y* magnetization of the FM contact is presented in Supplementary Figure 4, and the experimental and simulated signals are discussed in more detail in Supplementary Note 2.

**Figure 3:**
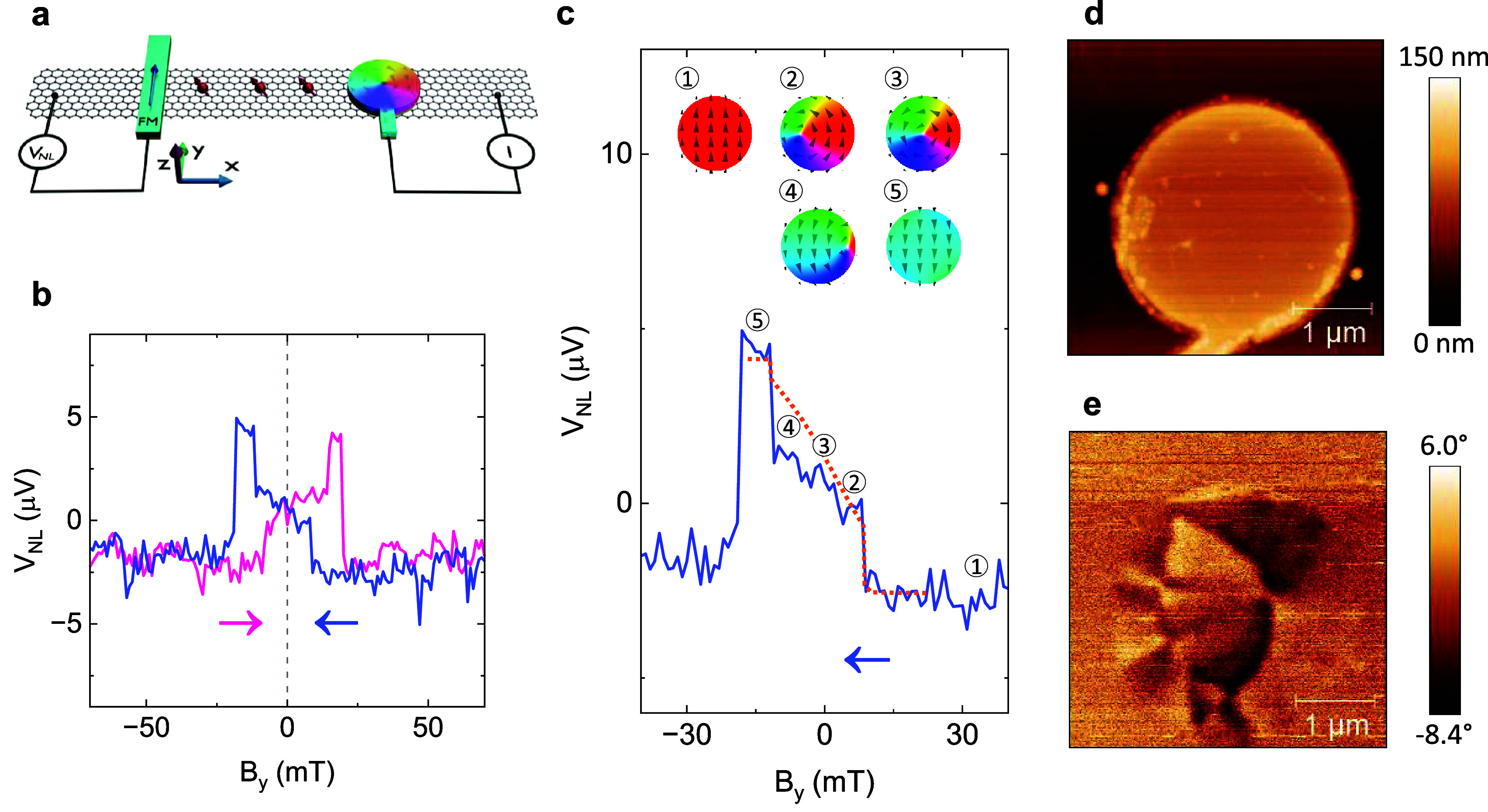
Detection of magnetic vortex textures in a circular microstructure. (a) Schematic of a spin-valve device with spin injection from a circular FM contact. (b) Measured spin-valve signal. The arrows indicate the *B*_*y*_ sweep directions. (c) Backward sweep of the spin-valve signal. The simulated *y* magnetization is indicated by a dotted line. Inset: Simulated magnetic textures illustrating the magnetization dynamics of the circular contact. The numbers 1–5 indicate the correlation between the magnetic textures and the measured spin-valve signal, where 3 is an intermediate point in the gradual shift between 2 and 4. (d, e) AFM topography (d) and MFM phase shift (e) images of a circular FM contact.

The magnetic texture of the circular contact was confirmed using MFM. The AFM topography and MFM images, where the phase shift correlates to the magnetization direction in the sample, are shown in Figure 3d,e. The main features in Figure 3e are dark and bright arcs that are extending outward from a point close to the center of the FM. This indicates a radial texture with changing magnetization around that point, such as a magnetic vortex. In fact, by generating an MFM image from a simulated vortex texture (shown in Supplementary Figure 4), similar dark and bright arcs could be replicated. The offset of the vortex core from the center of the contact is likely due to remanent magnetization from the in-plane field sweep. Discernibly, the experimental image also contains a smaller pattern toward the left side of the circular contact, which was not captured by the simulations. This may have arisen from inhomogeneities in the magnetic texture, such as smaller magnetic vortices, possibly because of defects in the cobalt or frustrations due to the relatively large size of the magnetic domains.^12^

Finally, a spin-valve device with a vortex spin texture in a **square** FM contact was measured, as illustrated in Figure 4a. The square contact has an ill-defined magnetic easy axis due to its 4-fold geometrical symmetry, but it is not fully symmetric like the circular contact.^11^ The measured spin-valve signal (Figure 4b) showcases sharp steps and gradual slopes with both increasing and decreasing *V*_*NL*_. This added complexity stems from the rectangular injector FM contact and the square detector FM contact (left and right in Figure 4a, respectively) having overlapping contributions to the measured spin signal. In order to alleviate the analysis of the spin-valve signal and the magnetization dynamics of the square contact, the contribution from the rectangular contact was subtracted. This was possible because the signal contribution of the rectangular contact is known from reference measurements (a single sharp step at the coercive field *B*_*y*_ = −19 mT; see Supplementary Note 3 for details). In this way, the contribution from the square contact could be isolated, as shown for the backward magnetic field sweep in Figure 4c.

**Figure 4:**
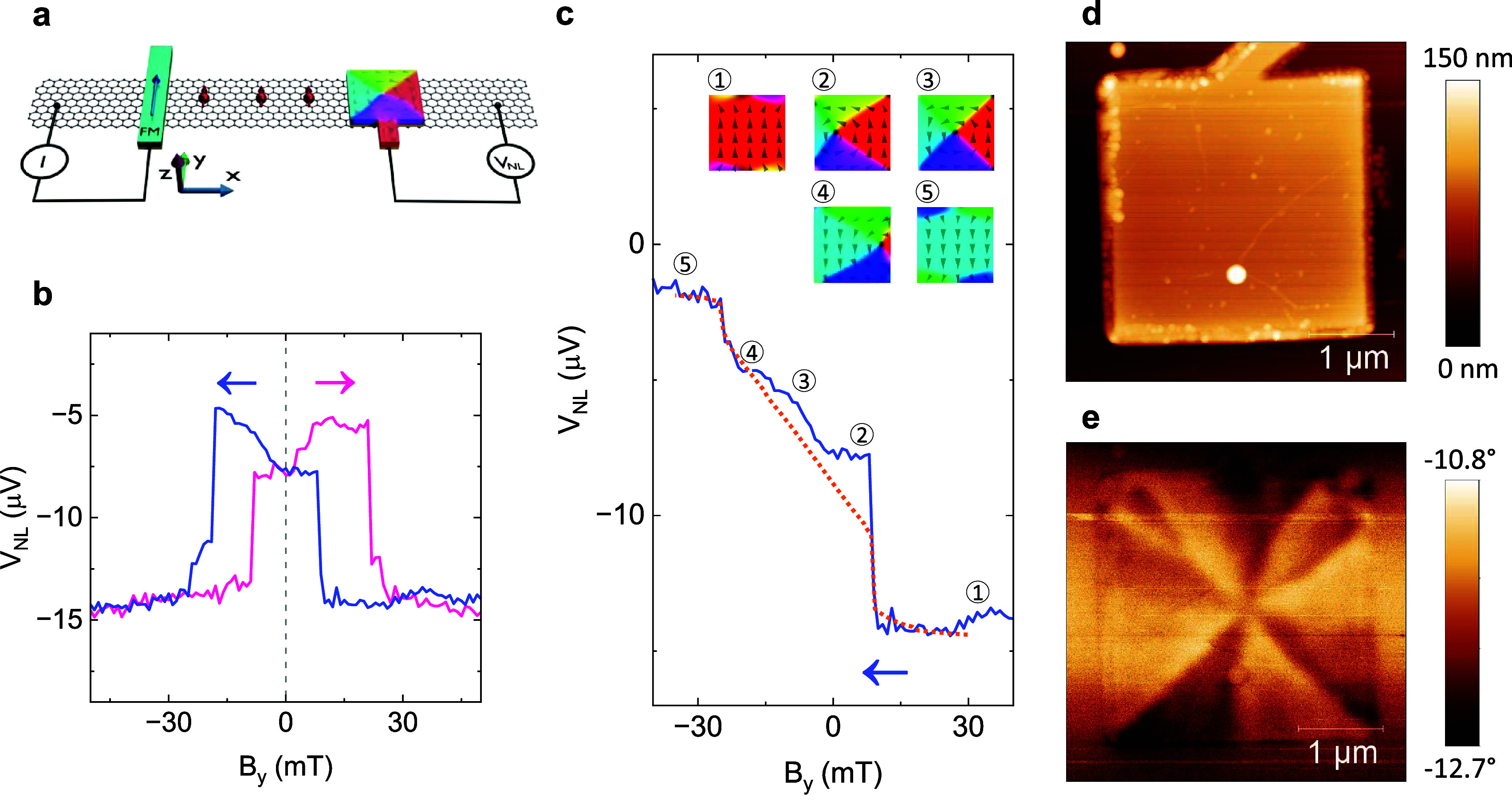
Detection of magnetic vortex textures in a square microstructure. (a) Schematic of a spin-valve device with spin detection with a square FM contact. (b) Measured spin-valve signal. The arrows indicate the *B*_*y*_ sweep directions. (c) Extracted spin-valve signal without the contribution from the rectangular injector contact, thus highlighting the contribution from the square contact. Only the backward sweep is shown for clarity. The simulated *y* magnetization is indicated by a dotted line. Inset: Simulated magnetic textures illustrating the magnetization dynamics of the square contact. The numbers 1–5 indicate the correlation between the magnetic textures and the spin-valve signal, where 3 is an intermediate point in the gradual shift between 2 and 4. (d, e) AFM topography (d) and MFM phase shift (e) images of a square FM contact.

The square contact contribution consists of a sharp step in *V*_*NL*_ before the applied field reaches zero, followed by a gradual slope and finally a second sharp step. Interestingly, the key difference between the circular and the square contact lies in the shape of the magnetization texture. When an external field *B*_*y*_ is swept from a large positive value toward zero, the magnetization of the square contact goes from being saturated in the +*y* direction to a vortex texture. However, this vortex texture is nonuniform and consists of four distinct triangular domains, contrary to the continuously changing spin orientations in the vortex texture of the circular contact.^12,54^ This special type of vortex is energetically preferential for the square contact since it encompasses both a flux-closing configuration and alignment of the spins with the straight contact edges.^52^ As the applied magnetic field is swept toward negative values, the vortex core moves continuously from the left side of the contact to the right side, after which it is eventually annihilated so that the magnetic texture becomes saturated with a −*y* alignment. These magnetization dynamics were also captured by simulations, which accurately reproduce both the expected magnetic textures (inset in Figure 4c and as an animation in Extended Data 2) and the shape of the experimental spin signal (Supplementary Figure 6). The minor deviations from the gradual slope of increasing *V*_*NL*_ may be attributed to pinning of the magnetic vortex by defects in the cobalt thin film.

The vortex dynamics in the square contact were also studied via minor-loop measurements (see Supplementary Note 4 for details), as an additional probe of the magnetic texture. Here, the *B*_*y*_ sweep was reversed before the annihilation of the magnetic vortex texture, leading to the spin signal retracing the gradual slope but not the sharp step in *V*_*NL*_. From this, it can be understood that the gradual slope and its associated continuous vortex movement are reversible, while the sharp steps and their associated vortex creation/annihilation events are hysteretic. This type of behavior is indeed expected for these types of magnetization dynamics, and acts as further support of our interpretation of the observed spin-valve signal. The AFM and MFM images in Figure 4d,e further confirm the existence of a nonuniform vortex texture in the square FM contact. Figure 4e shows an MFM image with a triangular pattern with straight borders between the bright and dark areas, noticeably different from the arcs in Figure 3e. A simulated magnetization texture with four distinct triangular domains gives rise to a near identical MFM image, as seen in Supplementary Figure 6, once again showing good agreement between experimental observations and theoretical calculations.

Finally, Hanle spin precession measurements were performed for some of the differently shaped FM contacts (see Supplementary Note 5 for details), where an applied out-of-plane magnetic field *B*_*z*_ caused the spins in the graphene channel to precess in the *xy* plane. Interestingly, the stepped FM contact generated similar spin signal amplitudes for the spin-valve and the Hanle spin precession measurements, while the square contact had a much smaller amplitude of the Hanle signal compared to the spin-valve signal, which indicates that the stepped contact was fully magnetized during the Hanle measurement, whereas the square contact had a vortex spin texture with a small net magnetization. Hence, this agrees well with the expected magnetic domains and textures from the spin-valve, MFM and micromagnetic simulation results above.

The magnetic domain formation and their dynamics with magnetic field sweeps can vary between different magnetic structures and also between different magnetic field sweeps. We would like to mention that, depending on the domain structures in the devices, the multilevel switching signals are reproducible, as demonstrated in Supplementary Figure 10–13, although switching fields can be different due to magnetic domain wall pinning effects. This includes the observation of single-step spin-valve signals in some instances where the geometric constrictions did not properly pin the domain wall to stabilize the intermediate magnetic states. Because of these effects, different spin-valve signal shapes were sometimes observed between forward and backward magnetic field sweeps, which is also present in conventional spin-valve devices. Hence, we find that our method has a good reliability for detecting spin textures, and that the aforementioned variability lies in the magnetization of the FM contacts, which can be improved through optimization of the fabrication process.

In conclusion, we demonstrated the electrical detection of a series of multidomain and vortex magnetic patterns at room temperature utilizing pure spin-polarized electronic transport in graphene spin-valve devices. The device allows for the discrimination of spin polarizations contributed by diverse domain patterns, resulting in distinctive multilevel spin-valve switching patterns. The validity of the different domain patterns in the investigated cobalt electrodes in the spin-valve devices was confirmed through magnetic force microscopy and micromagnetic simulations. These findings pave the way for exploring magnetic domain dynamics and spin textures using a pure spin current in graphene spin circuits at room temperature, including in two-dimensional (2D) magnets in all-2D heterostructures.^55,56^ Such technology can become pivotal for fast and energy-efficient read-out of magnetic states in innovative types of memory and computing applications, including racetrack^1,20^ and multilevel^3,5^ memories, compute-in-memory^10^ and neuromorphic computing^7,8^ devices.

## Methods

### Device Fabrication

The CVD graphene spintronics devices were fabricated on n++Si/SiO_2_ (285 nm) chips. The graphene channels were patterned from large-area CVD graphene (from Grolltex Inc.) to a width of 2–3 μm by electron beam lithography (EBL) and oxygen plasma etching. Characterization of CVD graphene channels is shown in Supplementary Figure 14. Both the NM (15 nm Ti/80 nm Au) and the FM (∼1 nm TiO_2_/60 nm Co) tunnel contacts were defined using EBL, followed by electron beam evaporation and lift-off processes. The FM contacts were produced by electron beam evaporation of Ti followed by *in situ* oxidation in a pure oxygen atmosphere to form a TiO_2_ tunnel barrier layer, after which Co was deposited in the same chamber. Our selection of scalable materials and fabrication processes is expected to alleviate future fabrication optimization and applications.

### Electrical Characterization and Measurements

The spin transport measurements were performed at room temperature in vacuum. A relatively low bias current (typically within −50 to −100 μA) was applied using a Keithley 6221 current source to minimize heating and spin-transfer-torque effects on the magnetic patterns. A negative current was used because this was found to maximize the signal-to-noise ratio in our setup (illustrated in Supplementary Figure 15), which can be due to hot-electron effects, magnetic proximity effects or electron-structure hybridization in the Co/graphene interface.^57^ The nonlocal voltage was detected by a Keithley 2182A nanovoltmeter. The magnetic field was applied with a GMW 5403 electromagnet. The three-terminal resistance of the FM contacts was generally in the range of 2–25 kΩ for the FM electrodes, and the sheet resistance of the graphene channels was around 1 kΩ/□.

### Micromagnetic Simulations

The micromagnetic simulations were performed in the GPU-based finite-difference micromagnetic software package MuMax3.^49–51^ Saturation magnetization *M*_*s*_ = 1325 kA/m, exchange stiffness *A*_*ex*_ = 20 pJ/m, Gilbert damping constant α = 2·10^–3^ and gyromagnetic ratio γ_0_ = 1.0·10^7^ Hz/T were used as parameters for all simulations. The FM electrodes were modeled by grids of 512 × 256 nanopillar cells with a cell size of 2 × 2 nm^2^ in the *xy* plane. The lateral extension of the grid was set to model a relatively large area of 8 × 4 μm^2^ (extending beyond the studied FM/graphene intersection area in order to avoid boundary-condition artifacts), which was scaled down to the aforementioned grid size to reduce calculation times. The magnetization of the FM/graphene intersection area was then extracted from the simulation results. The vertical dimension of the grid was typically set to either 1 × 60 nm or 2 × 30 nm. To be noted, the micromagnetic simulations in this work were only used for qualitative comparison with the experimental results, and refinement of the material parameters would be necessary for closer alignment with the experimental data. The MFM simulations were performed with the simulated magnetization textures as inputs and using the same magnetic parameters as above.

### AFM and MFM Measurements

AFM and MFM imaging was performed using a Bruker ICON AFM with a Nanoscope 6 controller in tapping and lift scan modes without an applied magnetic field. A Bruker MESP-V2MFM probe was used for both types of measurements. The MFM was measured using frequency contrast for the rectangular, stepped and notched FM contacts, and using phase shift for the circular and square FM contacts. Both frequency contrast and phase shift MFM images contain the same information about the second gradient of the magnetic force, which can be related to the magnetization direction, but the phase shift mode was found to give a slightly better image quality for the latter contacts, which is why it was used instead of the frequency contrast mode. The MFM measurements are discussed further in Supplementary Note 6.

Additional information about the devices and the presented measurement data is given in Supplementary Note 7.

## Supplementary Material







## Data Availability

The data supporting the findings of this study are available from the corresponding author upon a reasonable request.

## Notes

Additional experimental and micromagnetic simulation results; comment on discrepancies between experiments and simulations; detailed discussion about the vortex-dynamics spin-valve signals; isolating the spin-valve signal contribution from the square contact; minor-loop measurement for the square contact; Hanle spin precession measurements; notes on magnetic force microscopy; and notes on the measured devices (PDF)
Extended Data 1: magnetization dynamics animation of the circular FM contact (MP4)
Extended Data 2: magnetization dynamics animation of the square FM contact (MP4)

## References

[1] Parkin S. S., Hayashi M., Thomas L. (2008). Magnetic Domain-Wall
Racetrack Memory. Science.

[2] Gu K., Guan Y., Hazra B. K., Deniz H., Migliorini A., Zhang W., Parkin S. S. (2022). Three-dimensional
racetrack memory
devices designed from freestanding magnetic heterostructures. Nat. Nanotechnol..

[3] Chen S. (2024). All-electrical skyrmionic magnetic tunnel junction. Nature.

[4] Al
Bahri M., Sbiaa R. (2016). Geometrically pinned magnetic domain
wall for multi-bit per cell storage memory. Sci. Rep..

[5] Al
Bahri M., Borie B., Jin T. L., Sbiaa R., Kläui M., Piramanayagam S. N. (2019). Staggered Magnetic Nanowire Devices
for Effective Domain-Wall Pinning in Racetrack Memory. Physical Review Applied.

[6] Safranski C., Kaiser J., Trouilloud P., Hashemi P., Hu G., Sun J. Z. (2021). Demonstration of Nanosecond Operation in Stochastic
Magnetic Tunnel Junctions. Nano Lett..

[7] Ababei R. V., Ellis M. O., Vidamour I. T., Devadasan D. S., Allwood D. A., Vasilaki E., Hayward T. J. (2021). Neuromorphic
computation
with a single magnetic domain wall. Sci. Rep..

[8] Bunaiyan S., Datta S., Camsari K. Y. (2024). Heisenberg
machines with programmable
spin circuits. Physical Review Applied.

[9] Wang Q. (2020). A magnonic directional
coupler for integrated magnonic half-adders. Nature Electronics.

[10] Luo Z., Hrabec A., Dao T. P., Sala G., Finizio S., Feng J., Mayr S., Raabe J., Gambardella P., Heyderman L. J. (2020). Current-driven magnetic domain-wall logic. Nature.

[11] Cowburn R. P., Adeyeye A. O., Welland M. E. (1998). Configurational
anisotropy in nanomagnets. Phys. Rev. Lett..

[12] Shin Y.-S., Lee H.-J., Kim J., Park J., Char K. (2004). Magnetic domain
configuration in cobalt and permalloy micro-structures. Journal of the Korean Physical Society.

[13] Himeno A., Kasai S., Ono T. (2005). Current-driven domain-wall
motion
in magnetic wires with asymmetric notches. Appl.
Phys. Lett..

[14] Wachowiak A., Wiebe J., Bode M., Pietzsch O., Morgenstern M., Wiesendanger R. (2002). Direct observation
of internal spin structure of magnetic
vortex cores. Science.

[15] Schneider M., Hoffmann H., Otto S., Haug T., Zweck J. (2002). Stability
of magnetic vortices in flat submicron permalloy cylinders. J. Appl. Phys..

[16] Casola F., van der Sar T., Yacoby A. (2018). Probing condensed matter physics
with magnetometry based on nitrogen-vacancy centres in diamond. Nature Reviews Materials.

[17] Kläui M., Vaz C. A., Bland J. A., Wernsdorfer W., Faini G., Cambril E., Heyderman L. J., Nolting F., Rüdiger U. (2005). Controlled and reproducible domain
wall displacement by current pulses injected into ferromagnetic ring
structures. Phys. Rev. Lett..

[18] Trong
Hai N., Chen Z. T., Kindiak I., Bhatt R. C., Ye L. X., Wu T.-h., Zvezdin K. A., Horng L., Wu J. C. (2022). Electrical
characterization of magnetic domain wall via distinctive hysteresis
and magnetoresistance. J. Magn. Magn. Mater..

[19] Pham V. T., Zahnd G., Marty A., Savero Torres W., Jamet M., Noël P., Vila L., Attané J. P. (2016). Electrical
detection of magnetic domain walls by inverse and direct spin Hall
effect. Appl. Phys. Lett..

[20] Jeon J. C., Migliorini A., Yoon J., Jeong J., Parkin S. S. (2024). Multicore
memristor from electrically readable nanoscopic racetracks. Science.

[21] Zhao M. (2024). Electrical detection of mobile skyrmions with 100% tunneling magnetoresistance
in a racetrack-like device. npj Quantum Materials.

[22] Penthorn N. E., Hao X., Wang Z., Huai Y., Jiang H. W. (2019). Experimental Observation
of Single Skyrmion Signatures in a Magnetic Tunnel Junction. Phys. Rev. Lett..

[23] Raymenants E. (2021). Nanoscale domain wall devices with magnetic tunnel junction read
and write. Nature Electronics.

[24] Kimura T., Otani Y., Hamrle J. (2005). Determination
of magnetic vortex
chirality using lateral spin-valve geometry. Appl. Phys. Lett..

[25] Kimura T., Otani Y. (2007). Magnetization process
of a single magnetic ring detected by nonlocal
spin valve measurement. J. Appl. Phys..

[26] Ilgaz D., Nievendick J., Heyne L., Backes D., Rhensius J., Moore T. A., Niño M. A., Locatelli A., Menteş T. O., V. Schmidsfeld A., V. Bieren A., Krzyk S., Heyderman L. J., Kläui M. (2010). Domain-wall
depinning assisted by pure spin currents. Phys.
Rev. Lett..

[27] Motzko N., Burkhardt B., Richter N., Reeve R., Laczkowski P., Savero Torres W., Vila L., Attané J. P., Kläui M. (2013). Pure spin current-induced domain wall motion probed
by localized spin signal detection. Phys. Rev.
B.

[28] Avsar A., Ochoa H., Guinea F., Özyilmaz B., Van Wees B. J., Vera-Marun I. J. (2020). Colloquium:
Spintronics in graphene
and other two-dimensional materials. Rev. Mod.
Phys..

[29] Sierra J. F., Fabian J., Kawakami R. K., Roche S., Valenzuela S. O. (2021). Van der
Waals heterostructures for spintronics and opto-spintronics. Nat. Nanotechnol..

[30] Roche S. (2015). Graphene spintronics: The European Flagship perspective. 2D Materials.

[31] Tombros N., Jozsa C., Popinciuc M., Jonkman H. T., Van Wees B. J. (2007). Electronic
spin transport and spin precession in single graphene layers at room
temperature. Nature.

[32] Kamalakar M. V., Groenveld C., Dankert A., Dash S. P. (2015). Long distance spin
communication in chemical vapour deposited graphene. Nat. Commun..

[33] Khokhriakov D., Karpiak B., Hoque A. M., Dash S. P. (2020). Two-dimensional
spintronic circuit architectures on large scale graphene. Carbon.

[34] Panda J., Ramu M., Karis O., Sarkar T., Kamalakar M. V. (2020). Ultimate
spin currents in commercial chemical vapor deposited graphene. ACS Nano.

[35] Bisswanger T., Winter Z., Schmidt A., Volmer F., Watanabe K., Taniguchi T., Stampfer C., Beschoten B. (2022). CVD Bilayer
Graphene Spin Valves with 26 *μ*m Spin Diffusion
Length at Room Temperature. Nano Lett..

[36] Safeer C. K., Ingla-Aynés J., Herling F., Garcia J. H., Vila M., Ontoso N., Calvo M. R., Roche S., Hueso L. E., Casanova F. (2019). Room-Temperature
Spin Hall Effect in Graphene/MoS2
van der Waals Heterostructures. Nano Lett..

[37] Zhao B., Karpiak B., Khokhriakov D., Johansson A., Hoque A. M., Xu X., Jiang Y., Mertig I., Dash S. P. (2020). Unconventional Charge-Spin Conversion
in Weyl-Semimetal
WTe2. Adv. Mater..

[38] Hoque A. M., Sjöström L., Khokhriakov D., Zhao B., Dash S. P. (2024). Room temperature nonlocal detection
of charge-spin interconversion in a topological insulator. npj 2D Materials and Applications.

[39] Sierra J. F., Světlík J., Savero Torres W., Camosi L., Herling F., Guillet T., Xu K., Reparaz J. S., Marinova V., Dimitrov D., Valenzuela S. O. (2025). Room-temperature
anisotropic in-plane spin dynamics in graphene induced by PdSe2 proximity. Nat. Mater..

[40] Khokhriakov D., Sayed S., Hoque A. M., Karpiak B., Zhao B., Datta S., Dash S. P. (2022). Multifunctional
Spin Logic Operations
in Graphene Spin Circuits. Physical Review Applied.

[41] Wen H., Dery H., Amamou W., Zhu T., Lin Z., Shi J., Žutić I., Krivorotov I., Sham L. J., Kawakami R. K. (2016). Experimental Demonstration
of XOR
Operation in Graphene Magnetologic Gates at Room Temperature. Physical Review Applied.

[42] Finocchio G. (2024). Roadmap for unconventional computing with nanotechnology. Nano Futures.

[43] Selcuk K., Bunaiyan S., Singh N. S., Sayed S., Ganguly S., Finocchio G., Datta S., Camsari K. Y. (2024). Connecting physics
to systems with modular spin-circuits. npj Spintronics.

[44] Kläui M., Vaz C. A., Rothman J., Bland J. A., Wernsdorfer W., Faini G., Cambril E. (2003). Domain Wall
Pinning in Narrow Ferromagnetic
Ring Structures Probed by Magnetoresistance Measurements. Phys. Rev. Lett..

[45] Yuan H. Y., Wang X. R. (2014). Domain wall pinning in notched nanowires. Phys. Rev. B.

[46] Kamalakar M. V., Dankert A., Bergsten J., Ive T., Dash S. P. (2014). Enhanced
tunnel spin injection into graphene using chemical vapor deposited
hexagonal boron nitride. Sci. Rep..

[47] Dankert A., Venkata Kamalakar M., Wajid A., Patel R. S., Dash S. P. (2015). Tunnel
magnetoresistance with atomically thin two-dimensional hexagonal boron
nitride barriers. Nano Research.

[48] Kamalakar M. V., Dankert A., Kelly P. J., Dash S. P. (2016). Inversion of Spin
Signal and Spin Filtering in Ferromagnet |Hexagonal Boron Nitride-Graphene
van der Waals Heterostructures. Sci. Rep..

[49] Vansteenkiste A., Leliaert J., Dvornik M., Helsen M., Garcia-Sanchez F., van Waeyenberge B. (2014). The design
and verification of MuMax3. AIP Advances.

[50] Exl L., Bance S., Reichel F., Schrefl T., Peter Stimming H., Mauser N. J. (2014). LaBonte’s
method revisited: An effective steepest
descent method for micromagnetic energy minimization. J. Appl. Phys..

[51] Leliaert J., Van de Wiele B., Vansteenkiste A., Laurson L., Durin G., Dupré L., Van Waeyenberge B. (2014). Current-driven domain wall mobility
in polycrystalline Permalloy nanowires: A numerical study. J. Appl. Phys..

[52] Cowburn R.
P., Koltsov D. K., Adeyeye A. O., Welland M. E., Tricker D. M. (1999). Single-domain
circular nanomagnets. Phys. Rev. Lett..

[53] Antos R., Otani Y., Shibata J. (2008). Magnetic vortex
dynamics. J. Phys. Soc. Jpn..

[54] Van
Waeyenberge B., Puzic A., Stoll H., Chou K. W., Tyliszczak T., Hertel R., Fähnle M., Brückl H., Rott K., Reiss G., Neudecker I., Weiss D., Back C. H., Schütz G. (2006). Magnetic vortex
core reversal by excitation with short bursts of an alternating field. Nature.

[55] Zhao B., Ngaloy R., Ghosh S., Ershadrad S., Gupta R., Ali K., Hoque A. M., Karpiak B., Khokhriakov D., Polley C., Thiagarajan B., Kalaboukhov A., Svedlindh P., Sanyal B., Dash S. P. (2023). A Room-Temperature
Spin-Valve with van der Waals Ferromagnet Fe5GeTe2/Graphene Heterostructure. Adv. Mater..

[56] Ngaloy R., Zhao B., Ershadrad S., Gupta R., Davoudiniya M., Bainsla L., Sjöström L., Hoque M. A., Kalaboukhov A., Svedlindh P., Sanyal B., Dash S. P. (2024). Strong
In-Plane Magnetization and Spin Polarization in (Co0.15Fe0.85)­5GeTe2/Graphene
van der Waals Heterostructure Spin-Valve at Room Temperature. ACS Nano.

[57] Zhao B., Khokhriakov D., Karpiak B., Hoque A. M., Xu L., Shen L., Feng Y. P., Xu X., Jiang Y., Dash S. P. (2019). Electrically
controlled spin-switch and evolution of
Hanle spin precession in graphene. 2D Materials.

