# Supplementary material for: Electrical Detection of Magnetic Spin Textures Using Pure Spin Currents in Graphene
Source: Nano Lett. 2025 Dec 21;26(1):158–65. doi: 10.1021/acs.nanolett.5c04846 (PMC12810481; doi:10.1021/acs.nanolett.5c04846)
Supplement: Supplementary file 1 [file nl5c04846_si_001.pdf]

Supplementary information

Electrical detection of magnetic spin textures using pure spin currents in graphene

Lars Sjöström,[†] Bing Zhao,[†] Maha Khademi,[†,‡] Roselle Ngaloy,[†] Alexei Kalaboukhov,[†] Johan Åkerman,[¶,§,‖] and Saroj P. Dash[*,†,⊥,#]

†*Department of Microtechnology and Nanoscience, Chalmers University of Technology, SE-41296 Gothenburg, Sweden*

‡*NanOsc AB, SE-16440 Kista, Sweden*

¶*Department of Physics, University of Gothenburg, SE-41296 Gothenburg, Sweden*

§*Center for Science and Innovation in Spintronics, Tohoku University, 2-1-1 Katahira, Sendai 980-8577, Japan*

‖*Research Institute of Electrical Communication, Tohoku University, 2-1-1 Katahira, Sendai 980-8577, Japan*

⊥*Wallenberg Initiative Materials Science for Sustainability, Chalmers University of Technology, SE-41296 Gothenburg, Sweden*

#*Graphene Center, Chalmers University of Technology, SE-41296 Gothenburg, Sweden*

E-mail: saroj.dash@chalmers.se

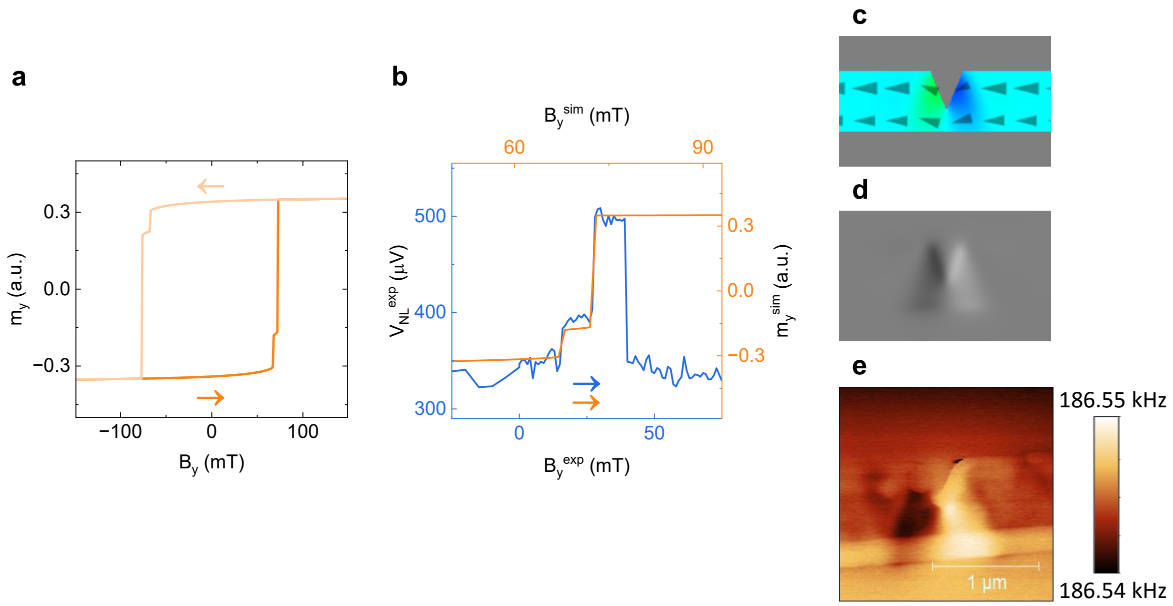

Supplementary Figure 1: Micromagnetic simulation of a single-notch contact. **(a)** Simulated hysteresis loop of a single-notch contact, showing the y magnetization of the ferromagnet (FM) as a function of external field B_y. The arrows indicate the B_y sweep directions. **(b)** Experimental spin-valve signal (blue) and simulated magnetization (orange) of a single-notch contact (same data as in Figure 2d of the main manuscript and in (a), respectively), showing qualitative agreement. **(c,d)** Simulated magnetization texture (c) and simulated MFM image (d) of the notch in a FM contact for zero applied field. **(e)** Experimental MFM frequency contrast image of a notch in a FM contact (also shown in Figure 2j of the main manuscript).

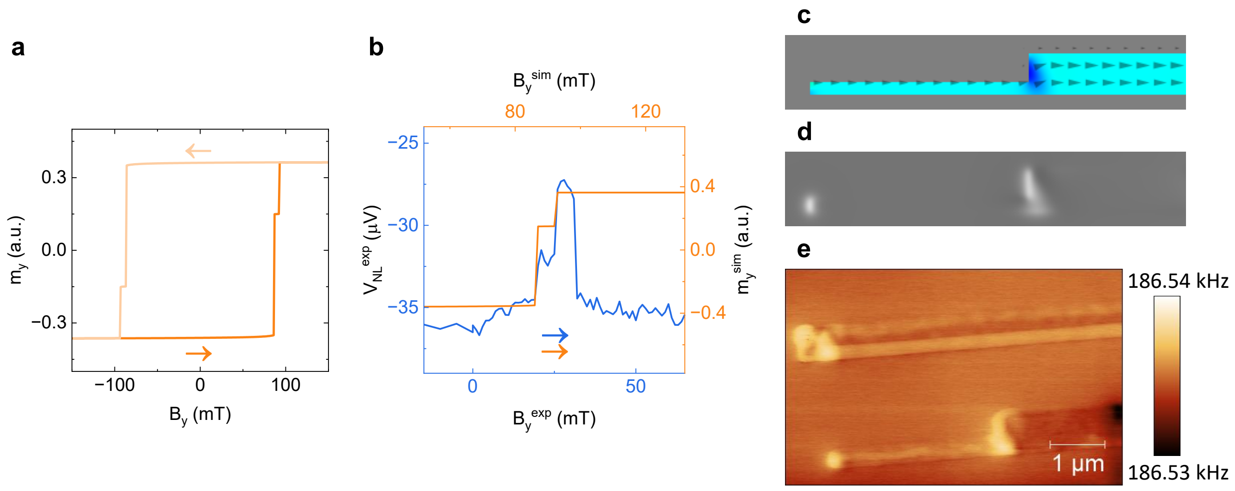

Supplementary Figure 2: Micromagnetic simulation of a stepped contact. **(a)** Simulated hysteresis loop of a stepped contact, showing the y magnetization of the FM as a function of external field B_y. The arrows indicate the B_y sweep directions. **(b)** Experimental spin-valve signal (blue) and simulated magnetization (orange) of a stepped contact (same data as in Figure 2h of the main manuscript and in (a), respectively), showing qualitative agreement. **(c,d)** Simulated magnetization texture (c) and simulated MFM image (d) of a stepped contact for zero applied field. **(e)** Experimental MFM frequency contrast image of a rectangular (top) and a stepped (bottom) FM contact (also shown in Figure 2l of the main manuscript).

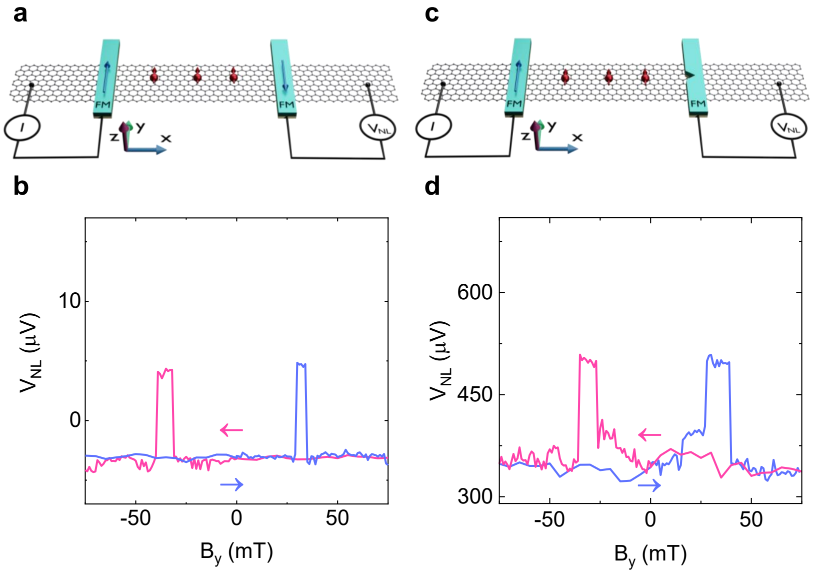

Supplementary Figure 3: Detection of multi-domain magnetic textures with forward and backward sweeps. **(a,c)** Schematics of spin-valve devices with two rectangular FM contacts (a) and with a single-notch FM contact (c). **(b,d)** Spin-valve signals with forward and backward magnetic field sweeps of the devices in (a,c), respectively. The blue curves are the same data as in Figure 2b,d of the main manuscript. The arrows indicate the B_y sweep directions.

Supplementary Note 1: Comment on discrepancies between experiments and simulations

Some quantitative discrepancies can be seen between the experimental spin-valve signals and the simulated hysteresis loops. This is especially evident in terms of the switching fields in Supplementary Figure 1b, 2b, 4b and 6b, where the magnetic switching happens at significantly larger magnetic fields in the simulations than in the experimental measurements.

One reason for the quantitative differences is that the simulations assume perfectly shaped homogeneous cobalt thin films, while there are unavoidable defects and other inhomogeneities in the real devices that can affect the magnetization textures and dynamics through pinning or other disturbances. Furthermore, material parameters such as saturation magnetization and exchange stiffness have not been investigated for the experimentally used cobalt thin films, which were deposited using electron beam evaporation, and it is possible that they deviate from the values that were used in the simulations.

Another likely source of discrepancy is the down-scaling of the simulation model compared to the physical systems, which is described in the Methods section of the main manuscript. While the down-scaling was necessary for reducing calculation times, the difference in length scales between the simulated model and the physical system is likely to have introduced some of the quantitative discrepancies between the simulated and the experimental results. Even though the dimensions of the simulated model are relatively small, the magnetization remains in the multi-domain regime, meaning that there are grain boundaries. The formation of these grain boundaries and the grain size are related to the size of the magnet, which in turn affects the coercive field due to domain wall motion. Importantly, however, we do not believe that the down-scaling has had any significant impact on the qualitative shapes of the simulated signals.

It should be emphasized that the simulations are only used for qualitative comparisons with the experimental results. Therefore, the aim with the simulations is not to reproduce quantitatively similar results compared to the experiments, but rather to correlate the qualitative signal shapes with different types of magnetization dynamics. Importantly, it is found that the switches and slopes in the experimental spin-valve signals correspond well with the expected magnetic domains and textures for the different types of FM contacts, as discussed in the main manuscript.

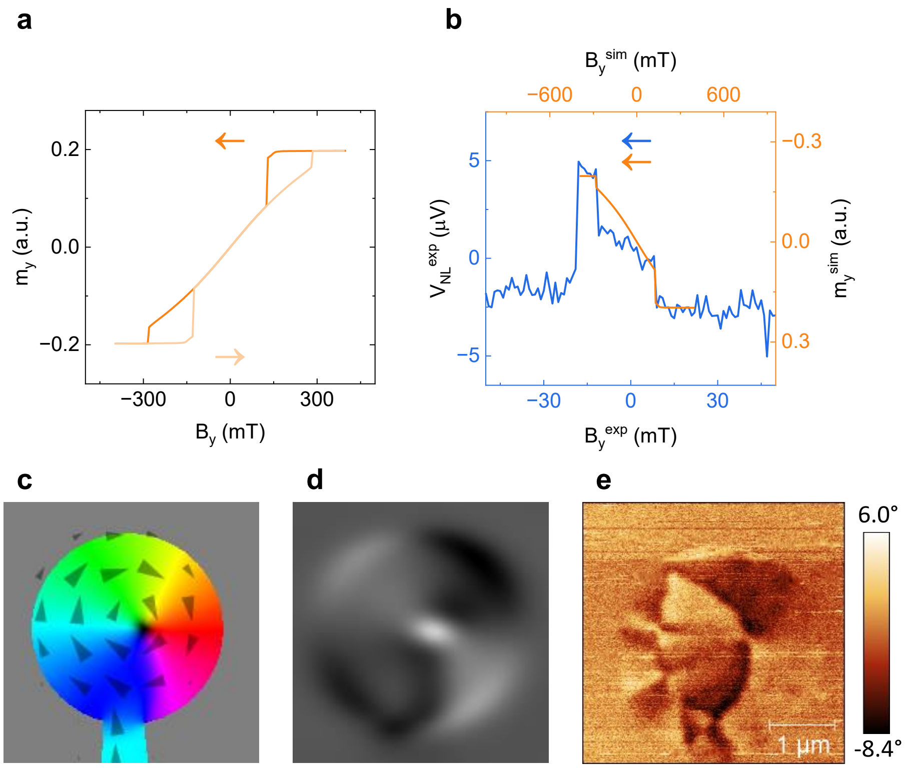

Supplementary Figure 4: Micromagnetic simulation of a circular contact. **(a)** Simulated hysteresis loop of a circular contact, showing the y magnetization of the FM as a function of external field B_y. The arrows indicate the B_y sweep directions. **(b)** Experimental spin-valve signal (blue) and simulated magnetization (orange) of a circular contact (same data as in Figure 3b of the main manuscript and in (a), respectively), showing qualitative agreement. **(c,d)** Simulated magnetization texture (c) and simulated MFM image (d) of a circular contact for zero applied field. **(e)** Experimental MFM phase shift image of a circular FM contact (also shown in Figure 3e of the main manuscript).

Supplementary Note 2: Detailed discussion about the vortex-dynamics spin-valve signals

A close examination of the spin-valve signals with vortex magnetization dynamics for the circular and square FM contacts shows some interesting details in terms of the step amplitudes. First, there is a difference in amplitude between the first (*i.e.* saturated-magnetization-to-vortex) and the second (*i.e.* vortex-to-saturated-magnetization) step, which is seen in the experimental spin-valve signal in Figure 4c of the main manuscript. Similarly, a difference between the first and the second step is also present in the micromagnetic simulations (as seen in Supplementary Figure 4a,b and 6a,b). The main cause for this asymmetry is concluded to be energy barriers between the different magnetic states, which shifts the switching fields towards the left (right) for the positive-to-negative (negative-to-positive) magnetic field sweeps. This shift is seen as hysteresis loops in Supplementary Figure 4a and 6a. Because of this, the magnetic vortex is closer to the center of the FM contact and has a smaller net magnetization immediately after its creation compared to immediately before its annihilation, which is captured by the simulated magnetic textures for point 2 and point 4 in the inset of Figure 3c and 4c of the main manuscript. Consequently, the change in net magnetization is larger during the creation event than during the annihilation event, which results in a larger step size in the spin-valve signal. This explanation is also consistent with the minor-loop observations in Supplementary Note 4.

Second, there is a discrepancy in step amplitude between the experimental spin-valve signals and the simulated magnetization curves, which is especially noticeable for the second step in Supplementary Figure 4b and for the first step in Supplementary Figure 6b. We ascribe this discrepancy to differences between the physical and the simulated systems, which is discussed in Supplementary Note 1. While we do not believe that this has had any significant impact on the qualitative shapes of the simulated signals, it is possible that some quantitative aspects such as the size of the steps may have been affected.

Supplementary Note 3: Isolating the spin-valve signal contribution from the square contact

The spin-valve signal from the measurements with a square ferromagnetic (FM) contact is complex, signifying intricate magnetization dynamics. Supplementary Figure 5c shows multiple sharp steps as well as gradual slopes. However, one of the sharp steps in the V_{NL} signal is expected to originate from the magnetization switching of the standard rectangular FM contact that the spin current is injected from. Hence, it is relevant to identify and remove the contribution from the standard FM contact, so that the signal contribution from the square contact can be extracted.

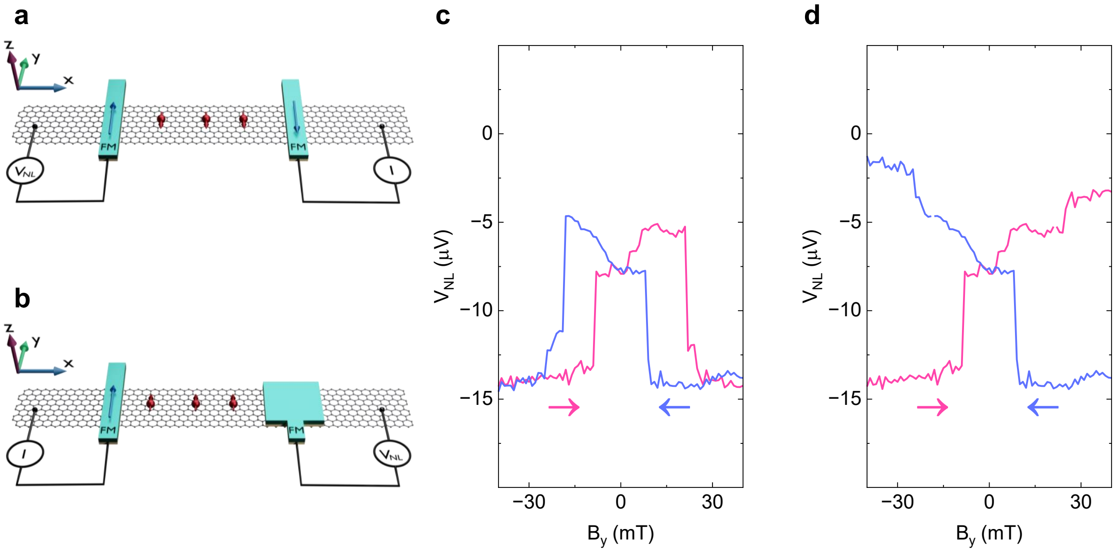

Supplementary Figure 5: Isolating the contribution from the square FM contact. (a) Schematic of a reference spin-valve device with two rectangular FM contacts. **(b)** Schematic of a spin-valve device with spin detection with a square FM contact. **(c)** Spin-valve signal measured using the device setup in (b) (same data as in Figure 4b of the main manuscript). The arrows indicate the B_y sweep directions. **(d)** The spin-valve signal from (c) without the contribution from the rectangular injector contact, highlighting the contribution from the square contact. The backward sweep is also shown in Figure 4c of the main manuscript.

Spin-valve signals from spin transport measurements between two standard FM contacts (Supplementary Figure 5a) and between one standard and one square FM contact (Supplementary Figure 5b), respectively, were compared in detail. Importantly, one of the standard FM contacts in the former measurement was used as the standard FM contact in the latter one. The contribution from this common FM contact is hence prevalent in both signals and could be identified as a single sharp step at $B_y = -19\,\mathrm{mT}$ ($B_y = 22\,\mathrm{mT}$) for the backward (forward) field sweep. In order to remove the impact from the standard FM contact, the signal component after the sharp step was translated and mirrored upside-down, resulting in Supplementary Figure 5d. This is necessary because the non-local voltage indicates the detected spin polarization with respect to the magnetization of the detector contact. When the magnetization of the spin injector contact (here, the standard FM contact) is reversed, the injected spins are also reversed and the detected spin signal gains a sign change. By

translating and mirroring the data points, this sign change is canceled. The remaining spin-valve signal, which corresponds to the magnetization from only the square FM contact, is discussed further in the main manuscript.

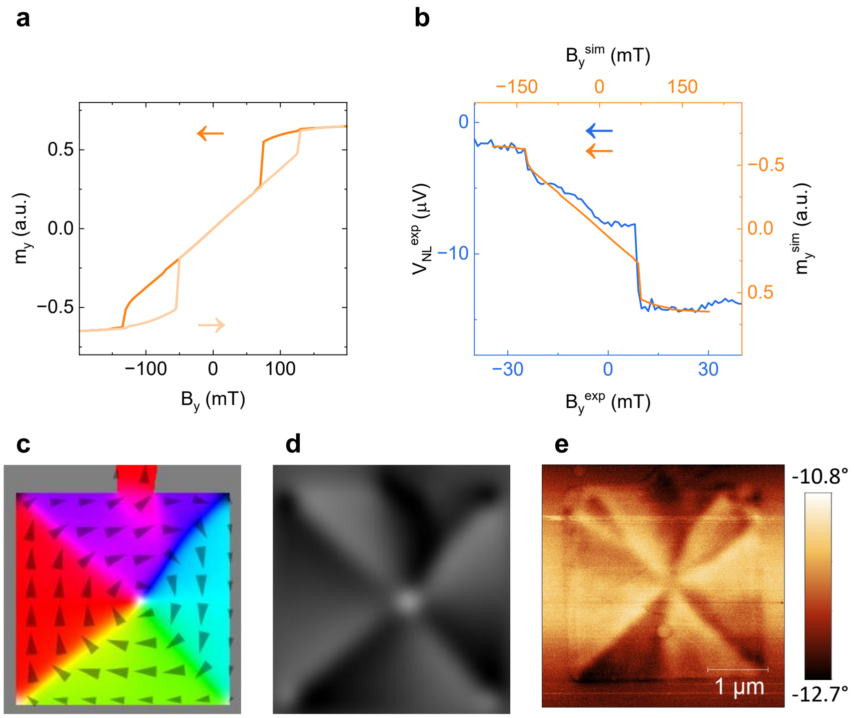

Supplementary Figure 6: Micromagnetic simulation of a square contact. **(a)** Simulated hysteresis loop of a square contact, showing the y magnetization of the FM as a function of external field B_y. The arrows indicate the B_y sweep directions. **(b)** Experimental spin-valve signal without the contribution from the rectangular FM contact (blue) and simulated magnetization (orange) of a square contact (same data as in Figure 4c of the main manuscript and in (a), respectively), showing qualitative agreement. **(c,d)** Simulated magnetization texture (c) and simulated MFM image (d) of a square contact for zero applied field. **(e)** Experimental MFM phase shift image of a square FM contact (also shown in Figure 4e of the main manuscript).

Supplementary Note 4: Minor-loop measurement for the square contact

The magnetization dynamics of the square FM contact were studied further through minor-loop measurements. In these measurements, the B_y sweep was reversed before the second sharp step in V_{NL} had been reached, which prevented the magnetization of the standard FM contact from switching (see Supplementary Note 3). The top curve in Supplementary Figure 7 shows that the gradual slope is reversible while the sharp step is hysteretic. This behavior is indeed expected, since the gradual slope is generated by the continuous movement of a magnetic vortex, which is a reversible process,[1] whereas the sharp steps are generated by the creation and annihilation of said vortex, which are hysteretic.[2,3]

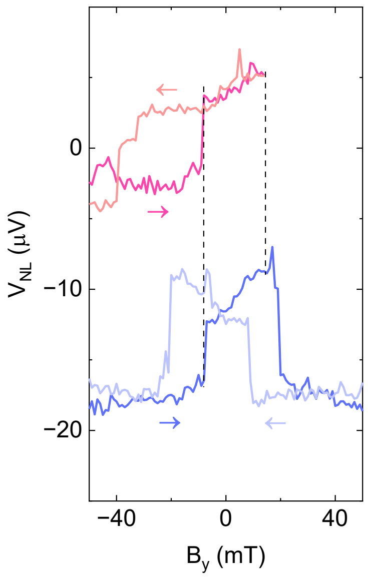

Supplementary Figure 7: Minor-loop measurement with the square FM contact. Minor-loop (top) and full-sweep (bottom) spin-valve measurements of the device in Supplementary Figure 5b. The arrows indicate the B_y sweep directions. The data has been shifted vertically for clarity. The field range of the reversible slope in V_{NL} is indicated by black dashed lines.

Supplementary Note 5: Hanle spin precession measurements

Hanle spin precession measurements were performed for some of the differently shaped FM contacts, where an applied out-of-plane magnetic field B_z causes the spins in the graphene channel to precess in the xy plane with a Larmor frequency that depends on the field strength, as illustrated schematically in Supplementary Figure 8a. A Hanle spin signal from spin transport between two standard rectangular FM contacts is shown in Supplementary Figure 8c. The experimental data was fitted by the spin diffusion and precession equation

$$V_{NL} \propto \int_0^{\infty} \frac{1}{\sqrt{4D_s t}} \mathrm{e}^{-L^2/4D_s t} \cos(\omega_L t) \mathrm{e}^{-t/\tau_s} \, \mathrm{d}t, \tag{1}$$

where D_s is the spin diffusion coefficient, τ_s is the spin lifetime, L is the length of the graphene channel and $\omega_L = g\mu_B B_z/\hbar$ is the Larmor frequency, where g is the Landé g-factor and μ_B is the Bohr magneton.[4,5] In this way, a spin lifetime of $\tau_s = 125 \pm 4\,\mathrm{ps}$ and a spin diffusion length of $\lambda_s = \sqrt{D_s \tau_s} = 1.72 \pm 0.07\,\mu\mathrm{m}$ could be extracted from the Hanle spin signal. Similar experiments with spin transport between one rectangular FM contact and one specially shaped FM contact yielded the Hanle curves in Supplementary Figure 8e,g for devices with a stepped and a square FM contact, respectively. The extracted spin transport parameters were $\tau_s = 53 \pm 3\,\mathrm{ps}$ and $\lambda_s = 1.06 \pm 0.03\,\mu\mathrm{m}$ for the stepped contact case, and $\tau_s = 135 \pm 29\,\mathrm{ps}$ and $\lambda_s = 1.23 \pm 0.13\,\mu\mathrm{m}$ for the square contact case. These parameter values can be considered as reasonably similar, and the variations between the three cases can originate from variations in graphene channel quality and contact properties.

The spin signal amplitudes of the spin-valve and the Hanle signals are compared in Supplementary Figure 9, where both signals are plotted together with the same scaling. Interestingly, a stark contrast can be seen between the cases with a stepped and with a square FM contact. For the stepped contact, the Hanle signal amplitude is roughly half that of the spin-valve signal (Supplementary Figure 9a), which signifies that the stepped contact is fully magnetized and contributes with a maximum in-plane spin polarization. For the square contact, on the other hand, the Hanle signal has a much smaller amplitude than the spin-valve signal, as shown in Supplementary Figure 9b. This indicates that the square contact has a small net in-plane magnetization during the Hanle measurement, which agrees well with the expected vortex spin texture for zero in-plane magnetic field.

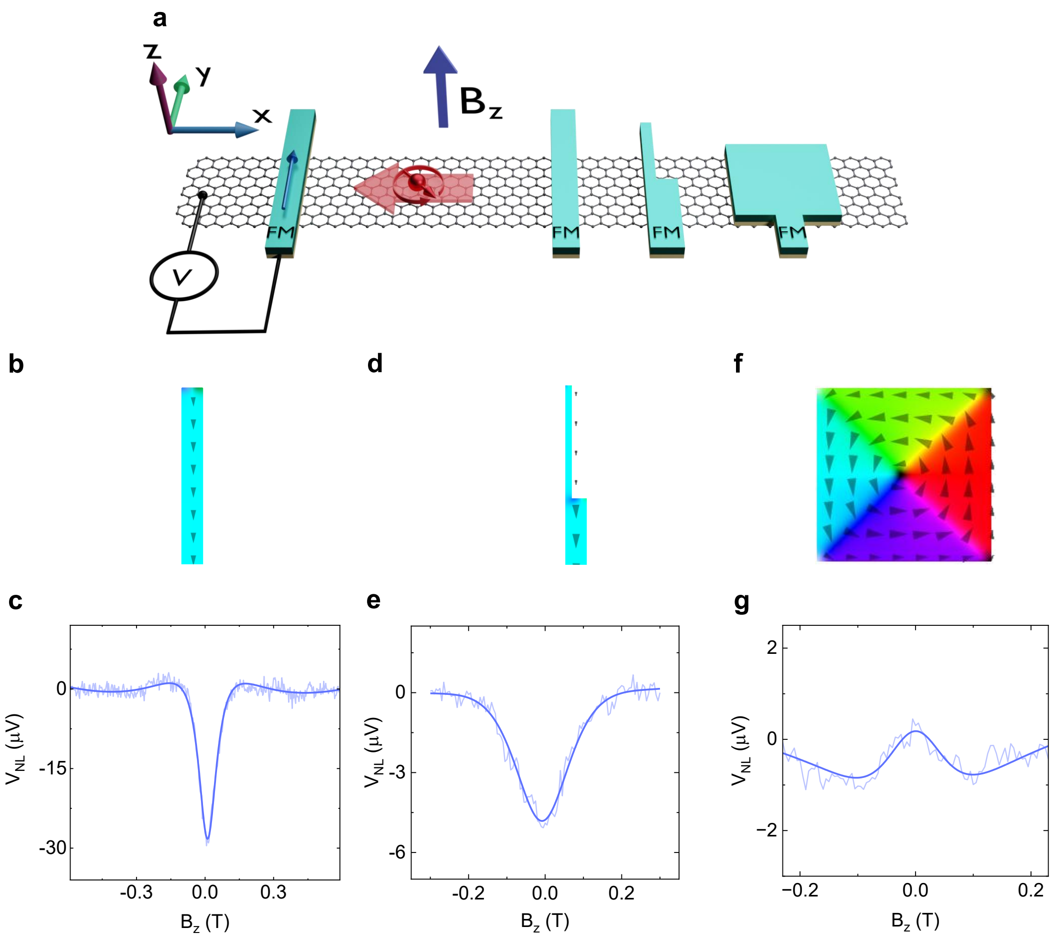

Supplementary Figure 8: Hanle spin precession measurements. **(a)** Schematic of Hanle spin precession in a spin-valve device with distinctly shaped FM contacts. To be noted, different devices and different reference contacts were used for the measurements. **(b,d,f)** Simulated magnetic textures of a rectangular (b), a stepped (d) and a square (f) FM contact, respectively, without an applied in-plane magnetic field. **(c,e,g)** Hanle spin precession signals of spin-valve devices with two rectangular FM contacts (c), a stepped contact (e) and a square contact (g), respectively. The dark blue curves are fits to the Hanle signals, using Equation (1).

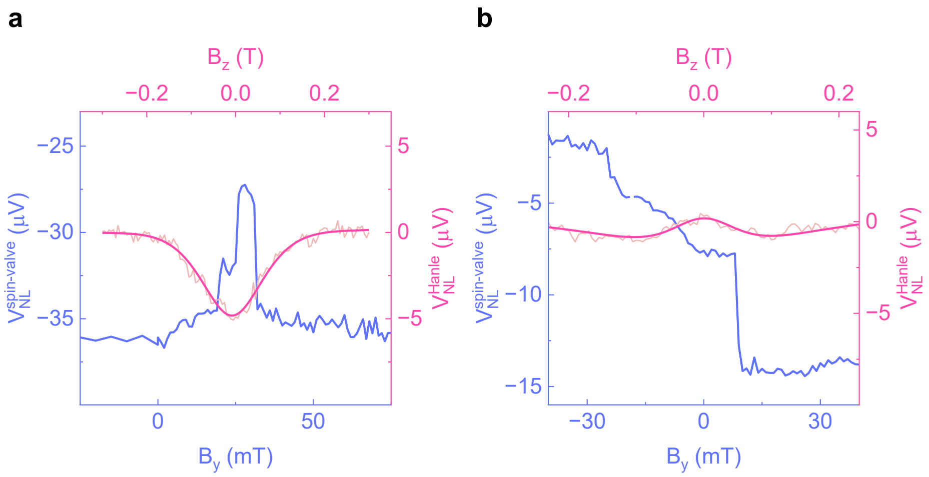

Supplementary Figure 9: Comparison between spin-valve and Hanle spin precession signals. (a,b) Spin-valve (blue) and Hanle spin precession (pink) signals from spin-valve devices with a stepped (a) and a square (b) FM contact, respectively, with the same scaling between the spin-valve and the Hanle signals (same data as in Figure 2h and Figure 4c of the main manuscript, and in Supplementary Figure 8e,g).

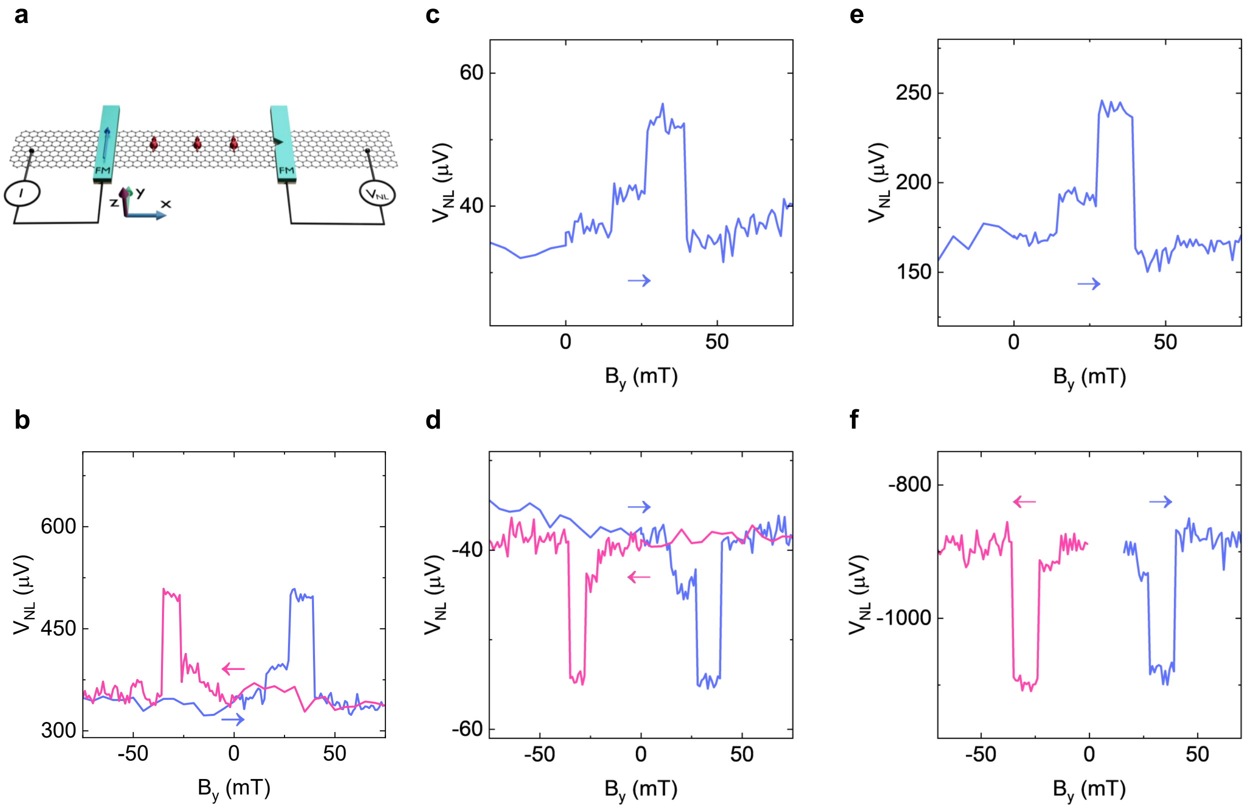

Supplementary Figure 10: Reproducibility of multi-domain switching in a notched contact. **(a)** Schematic of a spin-valve device with a single-notch FM contact. **(b-f)** Reproducible spin-valve signals from different magnetic field sweeps and different bias currents of the device in (a). (b) is the same data as in Supplementary Figure 3d. The arrows indicate the B_y sweep directions.

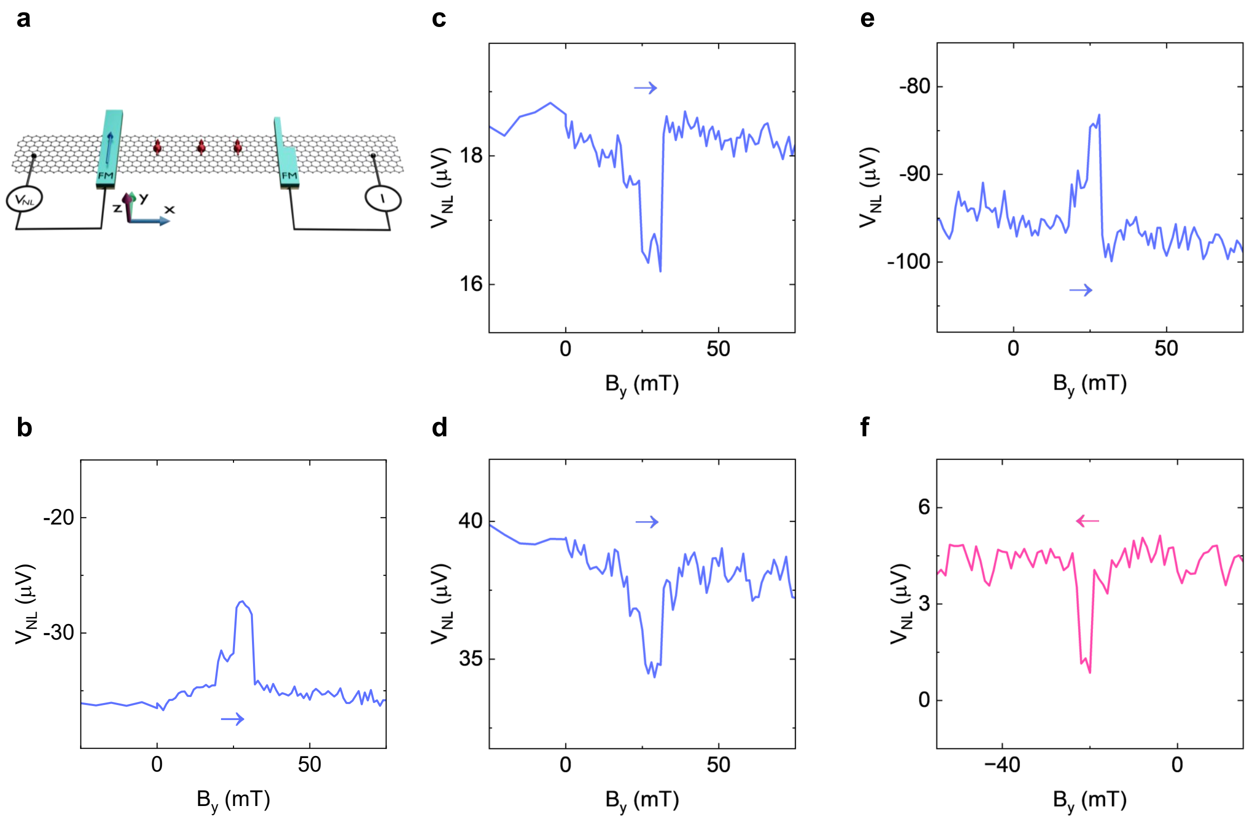

Supplementary Figure 11: Reproducibility of multi-domain switching in a stepped contact. **(a)** Schematic of a spin-valve device with a stepped FM contact. **(b-f)** Reproducible spin-valve signals from different magnetic field sweeps and different bias currents of different devices of the type in (a). (b) is the same data as in Figure 2h of the main manuscript. The arrows indicate the B_y sweep directions.

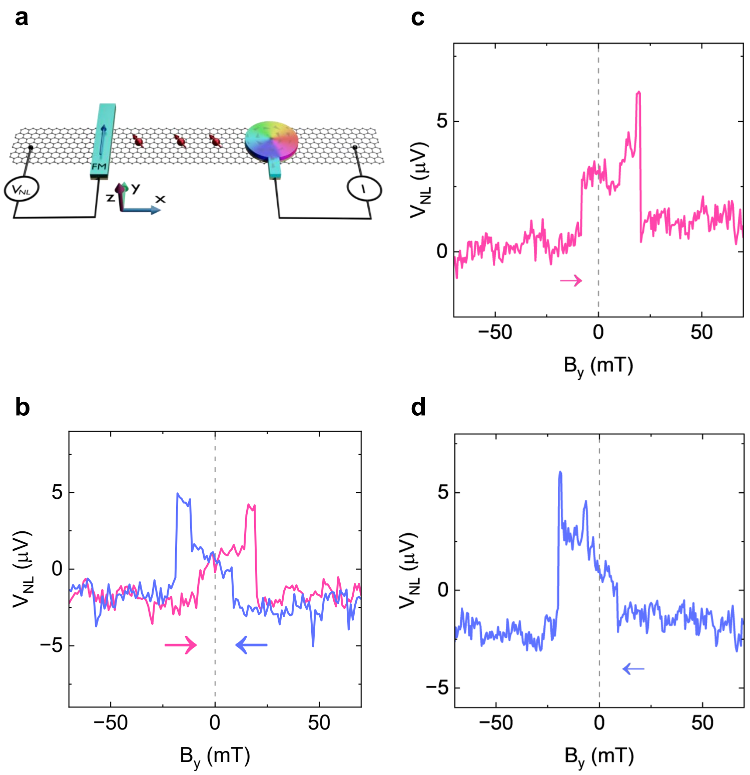

Supplementary Figure 12: Reproducibility of vortex switching in a circular contact. (a) Schematic of a spin-valve device with a circular FM contact. **(b-d)** Reproducible spin-valve signals from different magnetic field sweeps of the device in (a). (b) is the same data as in Figure 3b of the main manuscript. The arrows indicate the B_y sweep directions.

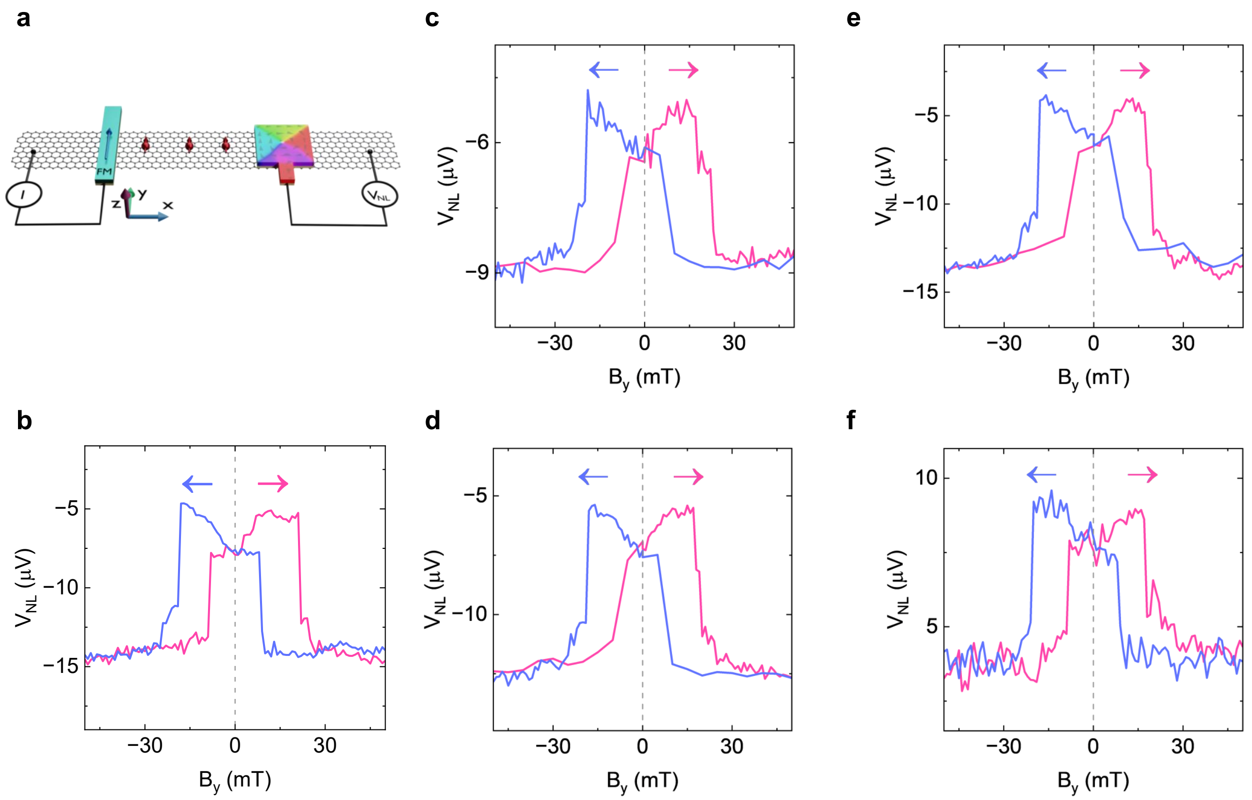

Supplementary Figure 13: Reproducibility of vortex switching in a square contact. (a) Schematic of a spin-valve device with a square FM contact. **(b-f)** Reproducible spin-valve signals from different magnetic field sweeps and different bias currents of the device in (a). (b) is the same data as in Figure 4b of the main manuscript. The arrows indicate the B_y sweep directions. The lower resolution of the signals before zero magnetic field in (c,d,e) is because fewer data points were collected in that region for these measurements.

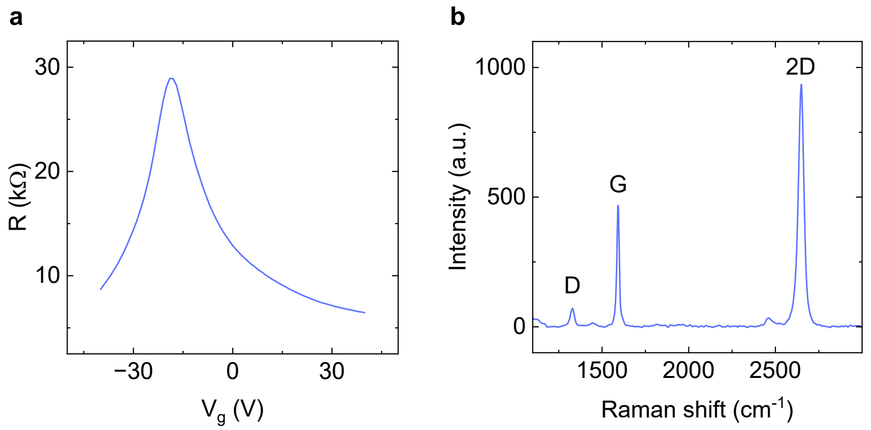

Supplementary Figure 14: Graphene characteristics. **(a)** Dirac curve of slightly doped chemical vapor deposition (CVD) graphene with a Dirac point of $V_D = -18\,\mathrm{V}$. The measurement was performed with $I = 0.5\,\mu\mathrm{A}$. **(b)** Raman spectrum of CVD graphene with pronounced G and $2D$ peaks and a small D peak.

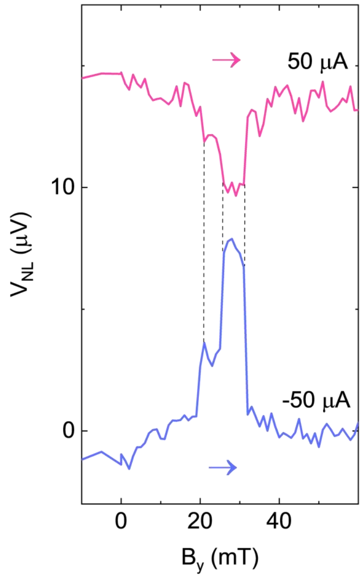

Supplementary Figure 15: Detection of multi-domain magnetic textures with positive and negative bias currents. Spin-valve signals from a stepped FM contact for positive (pink) and negative (blue) bias currents ($\pm 50\,\mu\mathrm{A}$). The arrows indicate the B_y sweep directions. The blue curve is the same data as in Figure 2h of the main manuscript and the pink curve is the same data as in Supplementary Figure 11d. The data has been shifted vertically for clarity. Both signals have very similar shapes with multi-step switching at the same magnetic fields (indicated by black dashed lines), but the negative bias current generates a spin signal with a slightly higher signal-to-noise ratio.

Supplementary Note 6: Magnetic force microscopy

The magnetic force microscopy (MFM) signal from the rectangular FM contact (shown in the top of Figure 2l of the main manuscript) is mostly homogeneous along the length of the contact, which confirms the quasi-single-domain nature of the contact's magnetization. A brighter contrast can be noted at the tip of the contact, which arises from stray fields, but this is outside of the graphene channel (see Supplementary Figure 16) and does not affect the spin-valve measurements. Similarly, this is also true for the brighter contrast at the tip of the stepped contact (shown in the bottom of Figure 2l of the main manuscript). Note, however, that the pinned magnetic domain wall is within the graphene channel area and has an impact on the measured spin signal, as discussed in the main manuscript.

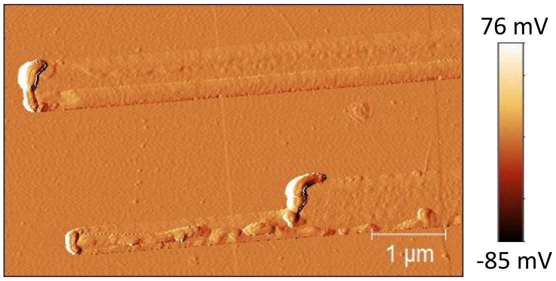

Supplementary Figure 16: AFM amplitude error image of rectangular and stepped contacts. Amplitude error image corresponding to the AFM image of the rectangular and stepped contacts in Figure 2l of the main manuscript. Here, the edges of the graphene channel are clearly visible, perpendicular to the FM contacts.

Through a comparison between the atomic force microscopy (AFM) and the MFM images in Figure 2i-l of the main manuscript, it can be noted that the dark and bright areas in the MFM images extend slightly beyond the physical shape of the FM contact. This can be understood as stray fields from the FM contacts that are detected by the MFM tip, which further confirms the magnetic origin of the signal.

Supplementary Note 7: Measured devices

The results in this work come from a couple of different devices, which contain differently shaped FM contacts. The reason for why multiple devices were used is that it is ideal to avoid additional cobalt electrodes on top of the spin transport channel, since the additional electrodes can disturb the spin transport through spin absorption.

The fabrication and measurement methodology are the same for all devices, as described in the Methods section of the main manuscript. Supplementary Table 1 lists the presented experimental data that was measured in each of the devices.

Supplementary Table 1: Device-figure correspondence. The measured devices of this work and the figures that contain the corresponding measurement data.

**Device**	**Figure**
Dev 1	Fig 1b, Fig 2d,h-l, Fig 4b-c, S Fig 3d, S Fig 7, S Fig 8e,g, S Fig 10c-f, S Fig 11c-e, S Fig 13c-f, S Fig 16
Dev 2	Fig 2f, S Fig 11f
Dev 3	Fig 3b-c, S Fig 12c-d
Dev 4	Fig 3d-e, Fig 4d-e
Dev 5	Fig 2b, S Fig 3b
Dev 6	S Fig 8c

Supplementary References

(1) Van Waeyenberge, B.; Puzic, A.; Stoll, H.; Chou, K. W.; Tyliszczak, T.; Hertel, R.; Fähnle, M.; Brückl, H.; Rott, K.; Reiss, G.; Neudecker, I.; Weiss, D.; Back, C. H.; Schütz, G. Magnetic vortex core reversal by excitation with short bursts of an alternating field. *Nature* **2006**, *444*, 461–464.

(2) Kimura, T.; Otani, Y.; Hamrle, J. Determination of magnetic vortex chirality using lateral spin-valve geometry. *Applied Physics Letters* **2005**, *87*, 172506.

(3) Cowburn, R. P.; Koltsov, D. K.; Adeyeye, A. O.; Welland, M. E.; Tricker, D. M. Single-domain circular nanomagnets. *Physical Review Letters* **1999**, *83*, 1042–1045.

(4) Han, W.; Kawakami, R. K.; Gmitra, M.; Fabian, J. Graphene spintronics. *Nature Nanotechnology* **2014**, *9*, 794–807.

(5) Khokhriakov, D.; Karpiak, B.; Hoque, A. M.; Dash, S. P. Two-dimensional spintronic circuit architectures on large scale graphene. *Carbon* **2020**, *161*, 892–899.
